# Prevalence of non-communicable diseases in Brazilian children: follow-up at school age of two Brazilian birth cohorts of the 1990's

**DOI:** 10.1186/1471-2458-11-486

**Published:** 2011-06-21

**Authors:** Antônio A Silva, Marco A Barbieri, Viviane C Cardoso, Rosângela F Batista, Vanda M Simões, Elcio O Vianna, Manoel R Gutierrez, Maria L Figueiredo, Nathalia A Silva, Thaís S Pereira, Juliana D Rodriguez, Sônia R Loureiro, Valdinar S Ribeiro, Heloisa Bettiol

**Affiliations:** 1 Departamento de Saúde Pública, Universidade Federal do Maranhão, São Luís, MA, Brasil; 2 Departamento de Puericultura e Pediatria, Faculdade de Medicina de Ribeirão Preto, Universidade de São Paulo, Ribeirão Preto, SP, Brasil; 3 Departamento de Medicina I, Universidade Federal do Maranhão, São Luís, MA, Brasil; 4 Departamento de Clínica Médica, Faculdade de Medicina de Ribeirão Preto, Universidade de São Paulo, Ribeirão Preto, SP, Brasil; 5 Departamento de Neurociências e Ciências do Comportamento, Faculdade de Medicina de Ribeirão Preto, Universidade de São Paulo, Ribeirão Preto, SP, Brasil; 6 Departamento de Medicina III, Universidade Federal do Maranhão, São Luís, MA, Brasil

## Abstract

**Background:**

Few cohort studies have been conducted in low and middle-income countries to investigate non-communicable diseases among school-aged children. This article aims to describe the methodology of two birth cohorts, started in 1994 in Ribeirão Preto (RP), a more developed city, and in 1997/98 in São Luís (SL), a less developed town.

**Methods:**

Prevalences of some non-communicable diseases during the first follow-up of these cohorts were estimated and compared. Data on singleton live births were obtained at birth (2858 in RP and 2443 in SL). The follow-up at school age was conducted in RP in 2004/05, when the children were 9-11 years old and in SL in 2005/06, when the children were 7-9 years old. Follow-up rates were 68.7% in RP (790 included) and 72.7% in SL (673 participants). The groups of low (<2500 g) and high (≥ 4250 g) birthweight were oversampled and estimates were corrected by weighting.

**Results:**

In the more developed city there was a higher percentage of non-nutritive sucking habits (69.1% vs 47.9%), lifetime bottle use (89.6% vs 68.3%), higher prevalence of primary headache in the last 15 days (27.9% vs 13.0%), higher positive skin tests for allergens (44.3% vs 25.3%) and higher prevalence of overweight (18.2% vs 3.6%), obesity (9.5% vs 1.8%) and hypertension (10.9% vs 4.6%). In the less developed city there was a larger percentage of children with below average cognitive function (28.9% vs 12.2%), mental health problems (47.4% vs 38.4%), depression (21.6% vs 6.0%) and underweight (5.8% vs 3.6%). There was no difference in the prevalence of bruxism, recurrent abdominal pain, asthma and bronchial hyperresponsiveness between cities.

**Conclusions:**

Some non-communicable diseases were highly prevalent, especially in the more developed city. Some high rates suggest that the burden of non-communicable diseases will be high in the future, especially mental health problems.

## Background

Cohort studies seek to verify and evaluate the health of the population and provide scientific data on the etiology of diseases, allowing the establishment of important preventive measures. These studies are increasingly used to answer many questions about health risks to the population due to their ability to generate new knowledge related to various outcomes [1].

Birth cohorts have the advantage of permitting the determination of health interferences suffered during the life cycle of an individual. They permit to understand the effects of exposures and experiences during intrauterine life and of childhood diseases on the future health of individuals, despite difficulty in obtaining data on exposure to early risk factors [2]. Classic examples are the studies of Barker, started in the 20th century, which related birth weight to mortality from chronic diseases in adults, such as cardiovascular disease and hypertension [3].

In Latin America there is an increased incidence of chronic non-communicable diseases [4]. However, many of the investigations of their possible etiologies, including studies conducted in Brazil [5], dealt only with risk factors acting in adulthood, following models of investigation such as the Framingham study [1].

In Brazil, the first longitudinal birth cohort study started in Ribeirão Preto, São Paulo State, in 1978/79. This research aimed to investigate the influence of socioeconomic conditions on perinatal health, human reproduction, infant mortality and medical care [6,7]. In 1982, the first birth cohort from Pelotas started and thereafter two other cohorts were initiated in 1993 and 2004 in this city [8]. In Ribeirão Preto, data from a new cohort of 1994 permitted comparisons with the 1978/79 cohort from the same city and with the 1997/98 São Luís cohort [9,10].

Prevalence rates of obesity and mental health problems in children are high and increasing[11,12]. Despite the growing number of cohort studies in progress to assess prevalence of non-communicable diseases in childhood[13], few have estimated the prevalence of depression, below average cognitive function, atopy and bronchial hyperresponsiveness, especially in low and middle-income countries. In Brazil there is also little information on regional inequalities in relation to these problems.

This article aims to describe the methodology of a school age follow-up of two birth cohorts initiated in 1994 in Ribeirão Preto and in 1997/98 in São Luís. It also aims to estimate and compare prevalences of some non-communicable diseases during the first follow-up of these cohorts. Ribeirão Preto is more developed than São Luís. Contrasting socioeconomic conditions are interesting to analyze in view of possible differences in the prevalence of different conditions, given that these cities are going through different stages of the epidemiological transition.

## Methods

### Study sites

The study was based on two birth cohorts, one initiated in Ribeirão Preto in 1994 and the other in São Luís in 1997-98, and on follow-up studies conducted in RP in 2004/05, when the children were 9-11 years old and in SL, in 2005/06, when the children were 7-9 years old. Ribeirão Preto is a city in the state of São Paulo, located in the Southeast, a rich and industrialized region. It had a population of 461,427 inhabitants in 1994[9]. Its Municipal Human Development Index was 0.855 in 2000[6]. São Luís is the capital of Maranhão state, located in the Northeast, one of the poorest regions of the country. It had a population of 781,068 inhabitants in 1997 [10]. Its Municipal Human Development Index was 0.778 in 2000[6].

### Assessment at birth

A total of 2923 infants born at 10 maternity hospitals of the city over a four-month period (April-August 1994) were included in the first stage of the Ribeirão Preto cohort study, representing 99% of all live births. Losses represented less than 5% of all births. Excluding twins, the final sample consisted of 2858 births to residents of the city [9].

The first phase of the São Luís cohort study was conducted from March 1997 to February 1998, using systematic sampling stratified by the number of births at 10 public and private maternity hospitals in the city. In each hospital one out of seven children was randomly selected, including 2541 hospital births (live births, stillbirths and singletons or multiple births of mothers living in the city). The sample was representative of births in the city because hospital births accounted for 96.3% of the total. After exclusion of multiple births (n = 50) and stillbirths (n = 48), the final sample consisted of 2443 births. Losses due to refusal or inability to locate the mother occurred in 5.8% of cases [10].

In the two cohorts, anthropometric data and information on pregnancy, childbirth and postpartum were collected at birth through a standardized questionnaire answered by the child's mother.

### Follow-up study of schoolchildren

Five birth weight groups were considered for the follow-up study: very low birth weight (VLBW <1500 g), low birth weight (LBW - 1500 to <2500 g), insufficient birth weight (2500 to <3000 g), normal birth weight (3000 to <4250 g), and children whose birth weight was at least two standard deviations above the population mean, who were classified as high birth weight (HBW ≥ 4250 g). The students in these weight groups with a smaller number of infants (VLBW, LBW and HBW) were oversampled in order to increase the study power.

**Figure 1:**
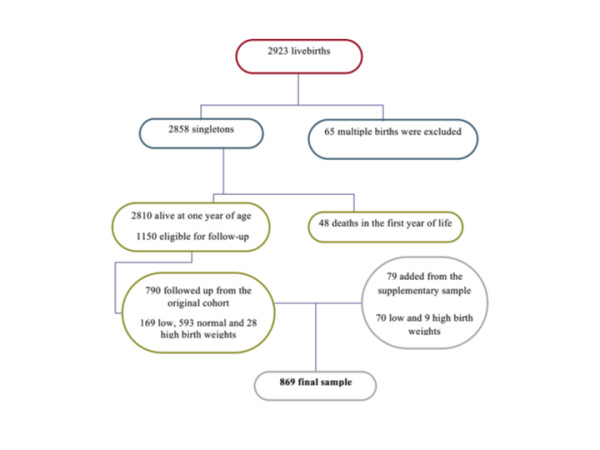
**Flow chart diagram of Ribeirão Preto birth cohort baseline and follow-up**.

**Figure 2:**
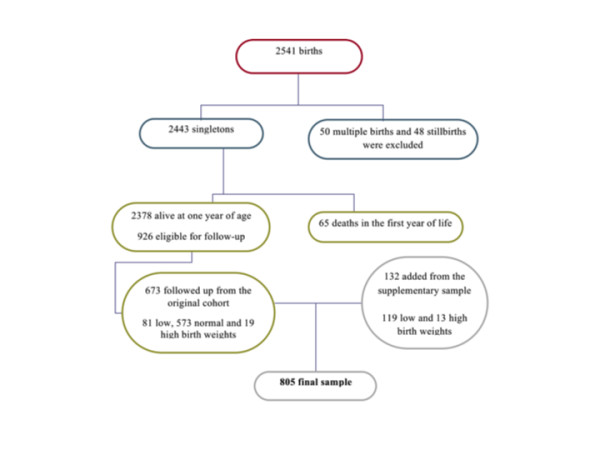
**Flow chart diagram of São Luís birth cohort baseline and follow-up**.

Children were traced at schools, or using the addresses supplied by the mother at birth and by media advertising. All parents or guardians of children in the groups of VLBW, LBW and HBW and a fraction of one out of three children in the groups of normal and insufficient birth weight were eligible and were invited to participate by telephone or mail. In Ribeirão Preto, after excluding deaths in the first year of life (n = 48), 1150 children were eligible for follow-up. The follow-up rate was 68.7% (169 LBW, 28 HBW and 593 normal and insufficient birth weight). A total of 790 children participated in the follow-up (Figure 1). In São Luís, excluding 65 deaths in the first year, 2378 children were alive at one year of age. Thus, 673 of 926 eligible children participated in the follow-up study (72.7% of the original target group, 81 being LBW, 19 HBW and 573 being of normal and insufficient birth weight) (Figure 2). Follow-up losses occurred due to migration, impossibility to locate the children, children not enrolled in school, and school or parental refusal.

### Additional sample

Since mortality was very high among children weighing less than 1500 g and ≥ 4250 g, a non-random additional sample was recruited, for a total of 132 children in SL and 79 in RP. Only children whose birth weights and gestational ages could be confirmed by inspecting hospital records or child health cards were included. Thus, the total sample included 1674 children, 869 from RP (Figure 1) and 805 from SL (Figure 2). The additional sample will only be included in selected studies to test hypotheses concerning groups of very low and high birth weight.

### Sample size and study power

A sample of 673 children (SL) and 790 (RP) has a 90% power to detect a minimum difference of 10% in the prevalence of the different conditions studied (estimated at about 50%, which is the prevalence that yields the maximum sample size) between the exposed and unexposed groups, with a 5% probability of type I error.

### Data Collection

A standardized questionnaire was applied to parents or guardians of children from the two cohorts, containing demographic questions and questions regarding general and oral health of the children. The questionnaire included variables concerning bruxism, nonnutritive sucking habits, recurrent abdominal pain and daily time spent in some sedentary activities. Cognitive function, mental health problems and childhood depression were also evaluated. Anthropometric examination was performed, and weight and height were measured. The questionnaire administered by the International Study of Asthma and Allergies in Childhood (ISAAC), translated and validated to assess asthma symptoms, was applied [14]. The assessment of asthma, bronchial hyperresponsiveness (methacholine challenge test) and skin tests for allergens was performed in a non-random subsample drawn by convenience. For each child of low birth weight two children of normal birth weight were included. The subsample included 328 children in RP and 330 in SL, corresponding to 41.5% and 49.0% of the original samples, respectively.

### Assessment of cognitive function

We used the Raven Colored Progressive Matrices Test, standardized for Brazilian children, which assesses general aspects of nonverbal intelligence, based on the multifactorial theory of Spearman [15]. It consists of five sets of 12 problems to infer the relationship between matrices. The sum of correct responses provides the overall score. It does not require reading and writing skills. The most satisfactory method of interpreting the overall score is by calculating the person's percentile by comparing his/her score to that attained by people of the same age and by identifying his/her relative position. This method makes no a priori assumption that the development of intellectual capacity is necessarily uniform or symmetrically distributed in childhood [15]. We calculated the prevalence of cognitive function below average or considered poor (≤ 25 percentile).

### Human figure drawing (HFD)

This is a maturity assessment that takes into account evolutionary and emotional indicators present in graphic production. It is also an indicator of cognitive development. It has been standardized by Wechsler in Brazil [16] and involves a simple activity - the drawing of a person using pencil and eraser. The standardized scores are converted to percentiles. We calculated the prevalence of below average, borderline or poor (below the 25th percentile) HFD.

### Mental health problems

The Strengths and Difficulties Questionnaire (SDQ) was proposed by Goodman in 1997 [17] for screening behavioral problems of children and adolescents between 4 and 15 years of age. It can be answered by parents/guardians, teachers or the adolescents themselves. In the present study a version of the questionnaire answered by parents was used. The questionnaire, aimed at identifying mental health problems, is composed of four subscales: emotional symptoms, conduct problems, hyperactivity/attention deficit, and peer problems. Scores are assigned to each of these scales (0, 1 or 2 as false, true or somewhat true). Then, a total score ranging from 0 to 40 is assigned at the end. According to the score, each scale is classified as normal, borderline or abnormal [17]. The presence of mental health problems was identified when the total score was greater than 16, which is considered abnormal [17]. The questionnaire was adapted and validated for Brazil [18].

### Childhood depression

It was measured by the Children's Depression Inventory (CDI). It is a screening instrument based on self-report prepared by Kovacs in 1981 which includes 27 items with three response options to be answered from 7 years of age onwards. The child selects the answer that best describes how he/she feels. It was adapted and validated by Gouveia *et al. *for Brazilian school children [19]. To each item 0, 1 or 2 responses are assigned, according to the order of the answers - 1st, 2nd or 3rd, respectively. Then the items are summed to obtain the total score. The score from which the child was considered to be "depressed" was less than 17 points, according to a Brazilian study [19].

### Bottle feeding, non-nutritive sucking habits and current bruxism

Parents were asked whether the child used a bottle, pacifier or sucked its finger ever in life. Finger sucking and pacifier use were considered non-nutritive sucking habits. Bruxism corresponded to the habit of grinding or clenching the teeth and was considered to be present if observed during sleep or when the child was awake; current bruxism was identified when parents or guardians reported that the habit had persisted until the day the interview took place.

### Primary headache

Primary headache was considered to be present when the mother reported that her child presented ≥ 2 episodes of headache in the two preceding weeks, without any associated organic symptoms, no matter how long or intense they were or where in the head they were located. This criterion was established according to the International Classification of Headache Disorders guidelines [20].

### Recurrent abdominal pain

Recurrent abdominal pain was considered to be the occurrence of pain or discomfort in the stomach in the last three months, strong enough to disrupt the child's daily activities such as playing, going to school or sleeping [21].

### Symptoms of asthma and bronchial hyperresponsiveness

The presence of symptoms of current asthma was investigated by adapting the questionnaire used in the asthma module of the ISAAC study [22]. The airway responsiveness was measured by the methacholine challenge test (bronchoconstrictor stimulus). The child inhaled methacholine at increasing concentrations, which started at 0.03 mg/ml and were successively doubled up to 16 mg/ml. The test was terminated when there was a decrease of 20% or more in forced expiratory volume in one second (FEV1) or when the last concentration of the protocol was reached. PC20, which is the concentration of methacholine causing a 20% fall in FEV1, was calculated. If PC 20 ≤ 2 mg/ml, the test was considered positive. Responsiveness was considered borderline if PC20 >2 to 16 mg/ml and normal if PC20 >16 mg/ml. For the examination the persons responsible for the children were instructed not to give the child tea, coffee, chocolate or soft drinks within 6 hours before the exam. To avoid false-positive results, the test was rescheduled if the child had had airway infection in the previous four weeks.

Asthma was considered to be present when subjective (symptoms of current asthma, or wheezing in the last 12 months) and objective criteria (presence of bronchial hyperresponsiveness) were present simultaneously or, by other definition, when subjective symptoms were reported alone.

### Skin tests for allergens

Skin tests for allergy were applied by the puncture technique by trained personnel. We used the following allergens: 3 dust mites (*Dermatophagoides pteronyssimus*, *Dermatophagoides farinae *and *Blomia tropicalis*), cat, dog, two types of cockroaches (*Periplaneta americana *and *Blatella German*), pollen and four fungi (*Alternaria alternata, Cladosporium herbarum, Aspergillus fumigatus *and *Penicillium notatum*). The result was considered positive when the wheal size was over 3 × 3 mm with hyperemia.

### Anthropometric and blood pressure measurements

Height and weight were measured, with the children barefoot and wearing light clothing, using standard techniques [23]. The instruments used were a precision scale calibrated periodically for measuring weight and an anthropometer for measuring standing height. For the assessment of nutritional status two references were used. The criteria proposed by the International Obesity Task Force (IOTF), based on data from six countries, including Brazil, used cutoffs of BMI for each age and sex, corresponding to cutoff points for overweight and obesity in adults [24]. For low weight the cutoff point <17 kg/m^2 ^was considered [25]. According to the American reference, children were considered underweight if BMI for age and gender was ≤ 5th percentile, overweight if BMI >P85 and ≤ P95 and obese when BMI > 95th percentile [26].

Blood pressure was measured three times with a digital sphygmomanometer, with cuffs adjusted for arm circumference. The measurements were performed by the same person, at 15-minute intervals with the participant resting in the sitting position, with his left arm at heart level. We calculated the average of the last two measurements. Hypertension was defined as systolic blood pressure and/or diastolic blood pressure equal to or greater than the 95th percentile for age, sex and height [27].

### Daily time spent in some sedentary activities

To assess the time spent in sedentary activities, the children reported the average daily time spent watching television, playing videogames or using the computer. Average daily time spent in some sedentary activities was divided into the following categories: 0 hours, up to 4 hours, more than 4 to 8 hours and over 8 hours.

### Other variables

The other variables considered were gender (male and female), child's skin color as reported by the mother/guardian (white, black, brown/mulatto and yellow/indigenous), monthly family income (<1, 1 to <4, 4 < 10 and 10 or more minimum wages), occupation of household head (non-manual, skilled manual and semi-skilled, unskilled/unemployed), maternal schooling in years (0-4, 5-8 and 9 or more years), maternal and paternal schooling in years (0-4, 5-8 and 9 or more years),performance of domestic chores by the child, and child labor (if the child perfomed some paid work outside the home, yes/no). For some variables, missing values were also presented.

### Statistical analysis and correction of estimates due to the complex sampling design

Due to the complex sampling design, children with birth weights <1500 g, 1500 g to 2499 g and ≥4250 g were overrepresented in the sample. Therefore, the prevalence estimates and their standard errors were weighted using the svy set of commands in Stata. The weighting took into account the different probabilities of selection of each of the five groups of birth weight and preterm birth. Stratification of the sample by birth weight was also taken into account. We used the chi-square test for comparison of proportions between the two cohorts and to assess differences in the percentage of follow-up according to maternal age, schooling and marital status, parity, and child's sex.

### Ethical Aspects

The reasons for the study and its methodological procedures were explained to the parents or guardians. After accepting the participation of their children in the research, they signed the consent form according to the guidelines and standards regulating research involving human subjects of the National Health Council, resolution 196/1996. Study participants were guaranteed the right to interrupt the study at any time, access to the results, confidentiality of the results, guidance and referral to specialized evaluation when necessary. The project was approved by the Research Ethics Committee of the University Hospital, Federal University of Maranhão (protocol number 3104-476/2005) and the Research Ethics Committee of the University Hospital, Faculty of Medicine of Ribeirão Preto, University of São Paulo (protocol number 6828/2004).

## Results

In RP, a lower percentage of children who participated in the follow-up study were born to cohabiting mothers, to mothers aged <20 years, with ≤ 4 years in comparison to eligible children who did not participate, but there was no difference regarding maternal parity and offspring sex. In SL, a lower proportion of children were born to mothers with ≥ 12 years of full time education, who were primiparous or gave birth to males in those who participated in the study in comparison to the eligible group who did not participate. However, there was no difference in the participants compared to non-participants regarding maternal age and marital status (Table 1). Differences in birth weight and preterm birth were observed due to the complex sampling design of the study and were corrected by weighting.

**Table 1:** Comparison of the characteristics of the participants at birth and school age.

**Variables**		**Ribeirão**	**Preto**				**São**	**Luís**		
		
	**n** **Initial sample (excluding 48 deaths)**	***n*** ***Eligible to follow-up***	***n*** ***followed up***	**% followed up**	p*	**n** **Initial sample (excluding 65 deaths)**	***n*** ***Eligible to follow-up***	***n*** ***followed up***	**% followed up**	p*
	
**Maternal age (years)**					0.005					0.350
20 to 34	2051	832	563	67.7		1,577	610	442	72.5	
≥35	265	114	94	82.5		101	41	32	78.0	
<20	487	202	131	64.9		698	274	199	72.6	
Missing	7	2	2	85.7		2	1	0	0.0	
**Marital status**					< 0.001					0.670
Married	1,664	666	489	73.5		695	266	199	74.8	
Cohabiting	690	291	158	54.2		1,107	437	314	71.9	
Single	338	147	106	72.1		575	223	160	71.7	
Missing	118	46	37	80.4		1	0	0	0.0	
**Maternal schooling (years)**					< 0.001					<0.001
≥ 12	367	145	93	64.3		119	46	14	30.4	
9 to 11	607	246	170	69.2		841	324	255	78.7	
5 to 8	1,028	416	304	73.1		1007	397	301	75.8	
≤ 4	618	267	158	59.1		405	157	103	65.6	
Missing	190	76	65	85.2		6	2	0	0.0	
**Parity**					0.361					0.049
1	1148	467	313	67.0		1156	457	316	69.1	
2 to 4	1474	600	423	70.5		1119	424	321	75.7	
≥ 5	160	73	46	63.0		103	45	36	80.0	
Missing	28	10	8	80.0		0	0	0	0.0	
**Sex**					0.714					0.001
Male	1425	581	402	69.2		1295	509	348	68.4	
Female	1384	569	388	68.2		1083	417	325	77.9	
Missing	1	0	0	0.0		0	0	0	0.0	
	
**Total**	2810	1150	790	68.7		2378	926	673	72.7	

Ribeirão Preto, 1994 and 2004/05 and São Luís, 1997/98 and 2005/06

* P value calculated by the chi-squared test.

In RP there was a predominance of white children, while children of mixed ethnicity prevailed in SL. RP showed higher proportions of children from families with higher family incomes and whose heads were engaged in non-manual occupations. More of the children from SL (95.7%) performed household chores than the children from RP (87.3%). A higher proportion of children whose mothers or fathers had formal education ≥ 9 years were seen in SL. Child labor was infrequent and did not vary between cities (Table 2).

**Table 2:** Socio-demographic factors, skin color of the child, performance of domestic chores by the child and child labor in the two birth cohorts.

Variables		Ribeirão Preto		São Luís	P value
	
	n	% weighted*	n	% weighted*	
**Sex**					0.724
Female	402	50.8	348	51.7	
Male	388	49.2	325	48.3	
**Skin color of the child**					<0.001
White	444	56.3	158	23.5	
Black	31	4.3	50	7.1	
Brown/Mulatto	304	37.9	463	69.1	
Yellow/Indigenous	11	1.5	2	0.3	
**Monthly family income (minimum wages)**					<0.001
< 1	36	4.5	149	23.0	
1 to <4	396	50.1	454	69.8	
4 to < 10	243	32.6	33	4.8	
≥ 10	103	12.8	19	2.5	
Missing	12	-	18	-	
**Occupation of household head**					<0.001
Non-manual	150	19.1	85	12.3	
Manual skilled/semiskilled	296	37.5	200	30.0	
Unskilled manual/unemployed	340	43.4	384	57.8	
Missing	4	-	4	-	
**Maternal schooling (years)**					0.001
0 to 4	345	43.9	223	34.4	
5 to 8	164	21.6	154	23.4	
≥ 9	273	34.5	278	42.2	
Missing	8	-	18	-	
**Paternal schooling (years)**					0.002
0 to 4	372	48.0	248	38.9	
5 to 8	148	19.5	128	20.3	
≥ 9	261	32.5	256	40.8	
Missing	9	-	41	-	
**Performance of domestic chores by the child**					<0.001
Yes	686	87.3	644	95.7	
No	104	12.7	27	4.4	
Missing	0	-	2	-	
**Child labor**					0.269
Yes	24	2.9	13	1.9	
No	766	97.2	659	98.1	
Missing	0	-	1	-	

Ribeirão Preto, 2004/05 and São Luís, 2005/06.

* Percentages may not add to 100% because of rounding. Missing values were excluded from percentages and P-value calculations.

The lifetime use of a bottle or pacifier was more frequent in RP than in SL. The number of children having the habit of finger sucking and clenching or grinding their teeth did not differ between the two cities. Mothers of children in RP reported more primary headache (27.9%) than in SL (13.0%). The prevalence of recurrent abdominal pain was similar in both cities, around 22% (Table 3).

**Table 3:** Lifetime bottle and pacifier use, non-nutritive sucking habits, current bruxism, primary headache and recurrent abdominal pain in the two birth cohorts.

Variables	Ribeirão Preto		São Luís		P value
		
	n	% weighted*	n	% weighted*	
**Lifetime bottle use**					< 0.001
Yes	709	89.6	463	68.3	
No	79	10.4	206	30.7	
Missing	2	-	4	-	
**Lifetime pacifier use**					< 0.001
Yes	502	63.8	263	38.7	
No	286	36.2	405	61.3	
Missing	2	-	5	-	
**Lifetime finger sucking**					0.137
Yes	80	9.9	80	12.4	
No	708	90.1	591	87.6	
Missing	2	-	2	-	
**Non-nutritive sucking habits (lifetime pacifier use or finger sucking)**					< 0.001
Yes	543	69.1	322	47.9	
No	243	30.9	346	52.1	
Missing	4	-	5	-	
**Current bruxism (teeth clenching or grinding)**					0.903
Yes	228	28.9	192	29.2	
No	551	71.1	472	70.8	
Missing	11	-	9	-	
**Primary headache (2 or more episodes in the two preceding weeks)**					< 0.001
Yes	206	27.9	87	13.0	
No	556	72.1	568	87.0	
Missing	28	-	18	-	
**Recurrent abdominal pain (pain in the last three months that affected the child's daily activities)**					0.887
Yes	172	21.9	144	21.6	
No	617	78.1	529	78.4	

Ribeirão Preto, 2004/05 and São Luís, 2005/06.

* Percentages may not add to 100% because of rounding. Missing values were excluded from percentages and P-value calculations.

Below average or poor cognitive function, whether assessed by the Raven test or the HFD test, was more prevalent in SL than in RP. Indicators of mental health problems according to the SDQ were also more prevalent in SL (47.4%) than in RP (38.4%). The prevalence of childhood depression was about three and a half times higher in SL (21.6%) than in RP (6%) (Table 4).

**Table 4:** Cognitive function, mental health problems and childhood depression in the two birth cohorts.

Variables		Ribeirão Preto		São Luís	P value
		
	n	% weighted*	n	% weighted*	
**Raven's test**					< 0.001
Below average or poor	103	12.2	202	28.9	
Average or above average	672	87.8	468	71.1	
Missing	15	-	3	-	
**Human Figure Drawing (HFD)**					< 0.001
Below average, borderline or poor	171	21.5	231	34.2	
Average or above average	603	78.5	435	65.8	
Missing	16	-	7	-	
**Strengths and Difficulties Questionnaire**					< 0.001
Abnormal	307	38.4	318	47.4	
Borderline	126	16.3	116	17.4	
Normal	351	45.2	236	35.2	
Missing	6	-	3	-	
**Children's Depression Inventory (CDI)**					< 0.001
Positive	56	6.0	141	21.6	
Negative	718	94.0	529	78.4	
Missing	16	-	3	-	

Ribeirão Preto, 2004/05 and São Luís, 2005/06.

* Percentages may not add to 100% because of rounding. Missing values were excluded from percentages and P-value calculations.

The prevalence of bronchial hyperresponsiveness as well as asthma was similar in both cities, both when considering only current symptoms or symptoms associated with bronchial hyperresponsiveness. RP had a higher percentage of children with positive skin tests to allergens (44.3%) than SL (25.3%) (Table 5).

**Table 5:** Asthma, bronchial hyperresponsiveness and atopy in the two birth cohorts.

Variables		Ribeirão Preto		São Luís	P value
		
	n	% weighted*	n	% weighted*	
**Wheezing in the last 12 months**					0.957
Yes	60	18.0	58	17.8	
No	268	82.0	270	82.2	
Missing	-	-	2	-	
Bronchial hyperresponsiveness **					0.069
Yes	135	39.6	111	32.5	
No	193	60.4	219	67.5	
Asthma ***					0.209
Yes	34	10.2	25	7.3	
No	294	89.8	305	92.7	
Atopy ****					< 0.001
Positive	142	44.3	81	25.3	
Negative	185	55.7	242	74.7	
Missing	1	-	7	-	

Ribeirão Preto, 2004/05 and São Luís, 2005/06.

* Percentages may not add to 100% because of rounding. Missing values were excluded from percentages and P-value calculations.

** PC20 ≤2 mg/ml

*** Asthma defined by a subjective criterion (wheezing in the last 12 months) plus an objective criterion (bronchial hyperresponsiveness)

**** Atopy was defined as at least one positive skin test for some allergens

The prevalence of overweight and obesity was higher than the prevalence of underweight among RP than SL children according to two criteria (Table 6). SL children spent more hours per day in sedentary activities (TV, videogames or computer) with an average of 5.6 hours per day, while in RP the average time spent in these activities was 4.6 hours per day (p < 0.001). The prevalence of hypertension in Ribeirão Preto (10.9%) was more than twice that observed in São Luís (4.6%). High systolic blood pressure was more than three times higher in Ribeirão Preto (9.3%) than in SL (2.5%). There were no differences in the prevalence of high diastolic blood pressure between the two cities (Table 6).

**Table 6:** Nutritional status, time spent in sedentary activities and arterial hypertension in the two birth cohorts.

Variables		Ribeirão Preto		São Luís	P value
		
	n	% weighted*	n	% weighted*	
**Nutritional status according to Cole *et al.***					< 0.001
Underweight	32	3.6	39	5.8	
Normal	542	68.6	595	88.8	
Overweight	139	18.2	25	3.6	
Obesity	75	9.5	13	1.8	
Unknown	2	-	1	-	
**Nutritional status according to Must *et al.***					< 0.001
Underweight	98	11.2	112	16.1	
Normal	492	63.4	522	78.5	
Overweight	104	13.5	24	3.5	
Obesity	94	11.9	14	1.9	
Unknown	2	-	1	-	
Time spent in sedentary activities (hours per day) **					< 0.001
0	13	2.0	6	1.0	
1 to 4	249	39.2	173	27.3	
> 4 to 8	371	57.0	399	60.1	
> 8	15	1.9	73	11.6	
Missing	142	-	22	-	
Systolic blood pressure ***					< 0.001
Normal	687	90.7	647	97.5	
High	69	9.3	18	2.5	
Missing	34	-	8		
Diastolic blood pressure ***					0.673
Normal	742	95.9	630	95.4	
High	29	4.1	30	4.6	
Missing	19	-	13		
Arterial hypertension ****					< 0.001
No	665	89.0	626	95.4	
Yes	79	10.9	31	4.6	
Missing	46	-	16	-	

Ribeirão Preto, 2004/05 and São Luís, 2005/06.

* Percentages may not add to 100% because of rounding. Missing values were excluded from percentages and P-value calculations.

** Daily time spent watching TV, playing videogames or on the computer

*** ≥P95 for age, sex and height

**** Systolic and/or diastolic blood pressure ≥P95 for age, sex and height

## Discussion

These two Brazilian cities are at different stages of the epidemiological transition. In the more developed city there was greater lifetime use of a bottle or pacifier, a greater prevalence of primary headache and atopy and a higher prevalence of overweight, obesity and hypertension. In the less developed city there was a higher percentage of children performing household chores, having lower than average cognitive function, mental health problems, depression and underweight. There was no difference between cities in the prevalence of bruxism, recurrent abdominal pain, asthma or bronchial hyperresponsiveness.

The prevalence of non-nutritive sucking habits was almost one and half times higher in RP than in SL. These prevalence rates are higher compared to studies conducted in rural (3%) [28] and urban areas (25.5%) [29] of India. The prevalence observed in SL was similar to that observed in Saudi Arabia (48.4%) [30] and in a Brazilian study (43.5%) [31]. The high prevalence of nonnutritive sucking habits observed suggests that the development of malocclusions will be frequent in these populations [32].

The current prevalence of bruxism was high in both cities, with no statistically significant difference between them. These prevalences were lower than those seen in Boston, 38% [33] and Canada, 45.6% [34].

The prevalence of primary headache, considered as the occurrence of two or more episodes in the last 15 days, in RP was more than twice that seen in SL. These high prevalence rates indicate that headache is an important public health problem among children. Frequent and severe headaches in the past year was reported by 17.1% of children and adolescents aged 4 to 18 years in the U.S. [35]. In Istanbul, 46.2% of the children reported headaches in the past year [36].

Prevalences of recurrent abdominal pain were high and did not differ between cities. Prevalence rates were higher than those reported in an international review, which ranged from 0.3% to 19% [21], indicating the need for further research on this neglected public health problem.

The higher prevalence of below average cognitive function in the less developed city by both the Raven test and the human figure drawing, probably reflects socioeconomic differences between the two cities, and differences in stardardization, since the benchmark for both tests was established with children from the Southeast. The high prevalence of mental health problems in this study, as measured by the SDQ was surprising. These prevalences were higher than those detected in studies in Germany (14.5%) [12] and Egypt (20.6%) [37]. Brazilian studies have found prevalence rates of 20.6% [38] and 24.6% [39], also lower than those detected in the present study. Methodological differences in these studies could not possibly justify such different rates. It is noteworthy that the SDQ tracks behavioral problems bud does not confirm the diagnosis.

The prevalence of childhood depression, as measured by the CDI was three and a half times greater in SL than in RP. The prevalence was high in SL, more than four times the prevalence of depression in children and adolescents aged 9-17 years, which has been estimated at 5% [40]. Using the CDI, a study conducted in Northern Ireland showed a prevalence of 11.6% in children aged 11 to 15 years [41]. In Brazil, the prevalence was 1.5% among children aged 7 to 14 years from a private school in Ribeirão Preto [42] and 13.9% among schoolchildren aged 7 to 13 years from a public school of Minas Gerais[40].

No statistically significant differences were observed between the two cities in asthma prevalence, which was around 18.0% when we considered the presence of wheezing in the past 12 months These figures are similar to those reported in the ISAAC study, in English speaking countries and in Latin America, in which the highest prevalence reported was 32.2%, also using as a criterion for the diagnosis of asthma wheezing within the past 12 months [43]. When we added an objective measure (the methacholine challenge test) these prevalence rates fell to about half (10.2% in RP and 7.3% in SL), suggesting that only using symptoms to diagnose asthma in epidemiological studies produces an overestimate of prevalence, due to the possible inclusion of false-positive results. The prevalence of bronchial hyperresponsiveness was high, considering a cutoff point of 2, and similar when comparing RP (39.6%) to SL (32.5%). Among children aged 7 to 10 years, the prevalence was 21.6% in the UK [44] and 28% in Boston [45], but those studies considered a cutoff point of 4.

Ribeirão Preto (44.3%) had a higher percentage of atopic children than São Luís (25.3%). In a study conducted in Porto Alegre on students aged 10 to 18 years, which used as a criterion for atopy at least one positive skin test, the prevalence was 50% [46], slightly higher than the values of RP and much more higher than the values of SL. In another study in China, also using a similar criterion for detecting atopy, prevalences ranged from 23.9% in Beijing to 41.2% in Hong Kong among schoolchildren aged 9 to 11 years, [47].

This difference in the prevalence of atopy between the two Brazilian cities may somehow be related to differences in lifestyle between developed and developing sites. It is thought that this phenomenon could also be related to differences in breastfeeding, smoking and infections. According to the hygiene hypothesis, in places with low transmission rates of pathogens, the immune system tends to shift to atopic responses [46]. However, based on this assumption, it would be expected that the prevalence rates of asthma and bronchial hyperresponsiveness would have been higher in the more developed city of RP, which was not the case.

The prevalence of obesity was higher in RP than in SL by two criteria. According to the IOTF, the prevalence of obesity in RP was 9.5%, lower than that observed in the United States in 2003-04 among children aged 6 to 11 years (18.8%) [48], but higher than the percentages observed in England in 2006/07 among children aged 8-10 years (5.6% to 6.3% for boys and girls) [49] and Rio de Janeiro in 1999 (5.0% for boys aged 7-14 years) [50]. The prevalence of obesity in SL (1.8%) was much lower than those observed in all the studies reported above. The prevalence of overweight in RP (18.2%) showed values similar to those of the United States (18.4%) [48], and England (19.8% to 23.9% for boys and girls) [49]. These results are consistent with studies showing the occurrence of the nutrition transition in developing countries. RP is experiencing an advanced stage of the nutrition transition, in which prevalence rates of overweight and obesity are higher than child malnutrition, while in SL child malnutrition rates (5.8%) are still higher than overweight (3.6%) and obesity rates (1.8%).

The average time spent in sedentary activities was greater in SL (5.6 hours per day) than in RP (4.6 hours per day). These results are consistent with national studies that show 3 to 5 h/day, on average, spent in sedentary activities [51,52], but a little above average hours found in international studies, which ranged from 2.2 to 4.5 h/day [53,54]. The children from SL spent more time watching TV and less time on videogames and computers, in contrast to from RP, where they spend less time watching TV and more time on electronic games (data not shown). This difference may be associated with the socioeconomic profile of the two cities, where availability of technology such as computers and videogames is greater for children in the more developed city.

The prevalence of arterial hypertension in RP was more than twice that observed in SL. International data show that the prevalence of hypertension ranged from 2.2% to 4.5% [55,56], The prevalence of hypertension in SL was similar to those observed in those studies, while in RP the prevalence was higher. In studies conducted in Brazil, the prevalence of hypertension ranged from 2.5% to 9.4% [57,58].

This study is one of the few population cohort studies conducted in developing countries that investigated non-communicable diseases among schoolchildren, contrasting a more developed with a less developed city. The strategy of oversampling LBW babies was used to increase the study power. Follow-up rates were satisfactory (68.7% in RP and 72.7% in SL).

In SL, children born to primiparous mothers with ≥12 years of schooling, or males had lower follow-up rates than their counterparts. In RP, participation rates were lower for children whose mothers lived in consensual union, had ≤ 4 years of full time education or were age < 20 years. It is possible that these differences might have led to an overestimation of some prevalence estimates in SL and to an underestimation in RP (eg, mental health problems), because groups of higher education were underrepresented in the former and overrepresented in the latter. The small age difference of two years was a limitation in comparing prevalence rates between the two cities. Another limitation is that prevalence estimates of asthma, bronchial hyperresponsiveness and atopy were obtained in a subsample drawn by convenience.

Differences in prevalence from other studies regarding the various indicators may have been due to real population differences in prevalence, differences in the age groups studied or differences regarding the methods of measurement, such as when assessing the prevalence of hypertension. In some studies used for comparison only one blood pressure measurement was taken, while in others two or three measurements were taken on one or more occasions.

## Conclusions

Some non-communicable diseases were highly prevalent among these Brazilian children, especially in the more developed city. Some high rates suggest that the burden of non-communicable diseases will be high in the future, especially mental health problems.

## Competing interests

The authors declare that they have no competing interests.

## Authors' contributions

AAS, MAB and HB conceived the study. AAS, TSP, MLF, NAS and MRG performed the statistical analysis. VCC, RFB, EOV, MRG, AAS, TSP, MLF, NAS, MRG, JDR, SRL and VSR analyzed the data. AAS, TSP, MLF, NAS, JDR, MAB, HB and wrote the paper. All authors read and approved the final version of the manuscript.

## Pre-publication history

The pre-publication history for this paper can be accessed here:

http://www.biomedcentral.com/1471-2458/11/486/prepub
